# Acceptability of virtual therapy for postpartum women during COVID-19: A national mixed methods study

**DOI:** 10.3389/fpsyt.2022.893073

**Published:** 2022-09-09

**Authors:** Carmen Gonzalez, Magaly Ramirez, Felicia Mata-Greve, Autumn Diaz, Miriana C. Duran, Morgan Johnson, Nancy Grote, Patricia A. Areán

**Affiliations:** ^1^Department of Communication, University of Washington, Seattle, WA, United States; ^2^Department of Health Services, School of Public Health, University of Washington, Seattle, WA, United States; ^3^Department of Psychiatry and Behavioral Sciences, School of Medicine, University of Washington, Seattle, WA, United States; ^4^School of Social Work, University of Washington, Seattle, WA, United States

**Keywords:** postpartum depression, virtual therapy, COVID-19, telehealth, digital health

## Abstract

**Background:**

Postpartum depression (PPD) affects one in eight women in the U.S., with rates increasing due to the COVID-19 pandemic. Given the unique circumstances of COVID-19, virtual therapy might be a unique way to overcome barriers to mental health services. The study sought to explore the acceptability of virtual therapy among women in the postpartum period.

**Methods:**

Using an online recruitment mixed methods approach, we collected data from a U.S. national cross-sectional sample of women (*N* = 479) who gave birth in the last 12 months.

**Findings:**

Results show that 66% of women endorsed items consistent with possible depression during the COVID-19 pandemic. Only 27% accessed therapy services during the postpartum period. While 88% were open to engaging in virtual therapy services, 12% identified several major concerns with virtual therapy, namely: (1) preference for in-person therapy (2) no perceived need for therapy (3) uncomfortable with virtual therapy, and (4) lack of privacy. Of note, 36% more Latinas reported dissatisfaction with quality of care received during virtual therapy compared to non-Latina participants. Despite a major shift to virtual care with COVID-19, future work is needed to make virtual mental health services more accessible for women with PPD.

## Introduction

Postpartum depression (PPD) is the most common pregnancy complication and affects approximately one in eight women in the U.S (1). Women of color and women from low-income backgrounds report even higher rates of PPD (2). With changes for many individuals due to mandated stay-at-home orders during the COVID-19 pandemic, rates of PPD have *doubled* compared to pre-pandemic rate (3). With the unique circumstances of the pandemic, digital mental health, or services delivered through technology platforms, might be a unique way to overcome barriers to care and effectively provide mental health support in the postpartum period. However, little is known about the acceptability of this form of care among postpartum women.

While postpartum mood disorders increase, there are still several barriers to postpartum mental health care as well as a shortage of providers that offer culturally appropriate services (4). For example, among 500 postpartum women from diverse backgrounds, only 4% of the sample indicated that they had no barriers to accessing mental health care (5). While many women report that common barriers to mental health care include lack of time, childcare, knowledge about PPD, stigma, and transportation concerns (4, 5), systemic inequities further affect treatment options available to women from low-income backgrounds (6), women of color (7), and women with limited English proficiency (4). Latina women often report being under- or uninsured as well as challenges with childcare, transportation, and leave (6, 8).

COVID-19 has underlined these disparities by increasing financial stress and social isolation for new parents. Home environments have also shifted during the pandemic, with mothers often taking on more care responsibilities as schools, businesses, and social services moved online (9). For postpartum women, the experience of giving birth and taking care of a newborn during a pandemic can lead to compounded trauma.

Digital mental health interventions have been proposed as a novel way to overcome barriers and shortages of postpartum mental health care. As a subset of digital mental health services, self-guided digital mental health apps have been developed and hold potential for reaching women with untreated PPD (10) as well as individuals negatively impacted by the COVID-19 pandemic (11). In fact, a recent systematic review of 21 experimental and randomized controlled trials found that perinatal women in high-income countries that used self-guided digital mental health apps and coach-guided digital mental health apps reported significantly decreased depression from pre- to post-intervention (12). These self-guided digital mental health apps might also contribute to the reduction of barriers related to transportation, childcare, time constraints, and language fluency for Latina women (13).

Fewer digital mental health services have used the feature of *virtual therapy*, or one-on-one contact between a patient and mental health provider in real-time (e.g., video conferencing, telepsychotherapy, and message-based care). Nair et al. (14) conducted a systematic review of 10 randomized controlled trials published between 2000 and 2018 to assess the effectiveness of virtual therapy, which included phone calls, emails, coach-guided mental health apps, and coach-guided websites to treat PPD. A majority of these studies reported significant improvement in depression scores post intervention, signaling promise for an understudied use of telemedicine. Virtual therapy can reduce barriers for postpartum mental health care, especially for women from underserved backgrounds, who are more likely to report difficulties accessing in-person therapy due to transportation, scheduling, and childcare concerns (4).

There are few studies that explore whether women in the postpartum period are *receptive* to virtual interventions (15). Outside of the postpartum literature, a recent study found that patients are open to virtual therapy and mental health apps with understandable apprehension surrounding privacy and quality of mental health care (16). Videoconferencing psychotherapy (VCP) specifically has been found to have similar outcomes to in person psychotherapy and is associated with positive user satisfaction (17). Thus, research is needed to understand the specific concerns of virtual therapy among women in the postpartum period, particularly during the COVID-19 pandemic and the rapid shift to telehealth.

The objectives of this mixed-methods cross-sectional study included: (a) to describe experiences with mental health services among women in the postpartum period during the COVID-19 pandemic, and (b) to explore the specific concerns of virtual therapy among women in the postpartum period. Because overwhelming evidence has demonstrated how COVID-19 has disproportionately affected Latinx populations in the U.S., we also examined how these experiences and concerns varied between Latina and non-Latina women (1). The findings from this study will inform how to address specific concerns of virtual therapy in future development of virtual mental health support.

## Methods

### Procedures

Participants completed the online cross-sectional survey between 7/16/20 and 8/28/20 through Prolific.co., an online platform that allows users to complete surveys and experiments (18). During the study period various states were still under stay at home, shelter in place, or social distancing orders that aimed to slow the spread of COVID-19. Survey participants included those that are: female, U.S. residents, with at least one child, 18–50 YO. In addition, participants completed an eligibility questionnaire to determine whether they had given birth in the last 12 months. Survey items were piloted and refined during five practice interviews. This study was reviewed and approved by an Institutional Review Board.

The survey took an average of 10 min to complete. Survey responses were captured using Research Electronic Data Capture (REDCap) (19, 20), a secure web application developed to capture data for research that complies with HIPAA standards. Prolific.co ID numbers were stored with the survey to avoid duplicate responses. Personal data were not collected with the exception of women who voluntarily provided their contact information (which was stored in a password protected computer at the home institution) for a follow-up interview. Participants received $5 compensation for their participation in the survey.

### Measures

#### Demographics

Participants completed a demographic questionnaire, which assessed age, assigned sex at birth, employment status, marital status, race, ethnicity, and English proficiency.

#### Experiences and beliefs about mental health services and virtual therapy

Survey questions were developed by the authors (experts in mental health, health services, health information and communication technology) to understand the experiences of women seeking mental health services in the postpartum period and their concerns with using virtual therapy during the COVID-19 pandemic. These questions asked about prior experiences with therapy, their willingness to consult a therapist, their preference for in-person or virtual therapy, and potential challenges to talking to a therapist online. Some items were open-ended (e.g., “Can you tell us a bit about your experience with receiving emotional support from a professional during your pregnancy?”) and some provided response options [e.g., “To what extent would you agree or disagree with the following statement: I am satisfied with the quality of care I received during virtual (therapy) interactions (strongly agree, agree, disagree, or strongly disagree)”].

#### Depression symptoms

Participants completed the Edinburgh Postnatal Depression Scale (EPDS) (21, 22), a 10-item questionnaire that screens for PPD. Participants indicate whether they have experienced symptoms of depression (e.g., “I have blamed myself unnecessarily when things went wrong”). Summary scores are calculated (range from 0 to 30). We categorized respondents into four categories based on scoring guidelines outlined by Cox et al. (21): depression not likely (<8), depression possible (9–11), fairly high possibility of depression (12–13), and probable depression (14 and higher). We grouped women into depression not likely (score of 8 or below) and depression possible (score of 9 or above). In the current study, internal consistency reliability was excellent (Cronbach α = 0.90; omega = 0.90). Participants who reported suicidality were sent a follow up email with a list of postpartum and suicide prevention resources.

### Analysis

#### Quantitative data analysis

Means and frequencies were used to document demographic information, postpartum mental health outcomes, experiences with mental health services in the postpartum period, and concerns with virtual therapy. Percentages reported in the results do not reflect missingness (*N* = 479). To further explore how COVID-19 has disproportionately affected the Latinx population in the U.S., we assessed for differences in experiences and concerns between Latina and non-Latina participants using Fisher's exact chi-square tests, chi-square tests, and independent *t*-tests. We only present results when there is a statistically significant difference between Latina and non-Latina participants. All quantitative statistical analyses were performed with SAS version 9.4.

#### Qualitative data analysis

We conducted a thematic analysis of the responses to an open-ended question about reasons why participants would not be open to consulting a therapist virtually. Textual responses to the open-ended questions varied from a few words to a few sentences, with participants writing an average of 50 words. For theme identification, we first used an inductive approach that involved two authors pile-sorting each participants' response into categories based on affinity (i.e., the reason for why they were not interested in virtual therapy). Affinity diagramming, a user-centered design approach, is an exploratory qualitative analysis method that facilitates the creation of a codebook; a pile sort is a grouping of similar thoughts, perspectives, or statements (23). This process identified four salient categories: (1) preference for in-person therapy, (2) no perceived need for therapy, (3) not comfortable speaking to a therapist, and (4) lack of privacy at home. To identify subthemes, the two authors further pile-sorted responses within each of the four categories. We provide a description of each category and include representative quotes from participants' responses. All qualitative analyses were performed using Dedoose coding software.

## Results

### Participant characteristics

See Table 1 for characteristics of study participants. There were 2416 individuals who were interested in completing the survey. In total, 479 women were screened eligible and completed the survey. The average age was 30.3 years. Most (72%) of the sample identified as White, and ~7% of the sample identified as Latina. Most participants (97%) indicated that they spoke English “very well.” Participants reported residing in state across the U.S., with larger states such as California, Florida, and New York more highly represented. Approximately two-thirds of the sample were in the “depression possible” range for PPD.

**Table 1:** Sample characteristics.

**Characteristic**	**Total (*N* = 479)**
**Age**	
*N*	474
Mean (*SD*)	30.3 (5.1)
**Race**, ***n*** **(%)**	
Black or African American	60 (12.5%)
Hispanic or latino/x	22 (4.6%)
White or caucasian	344 (71.8%)
Asian or pacific islander	24 (5.0%)
Multiracial	24 (5.0%)
Unlisted	5 (1.0%)
**Hispanic or Latina**^**a**^, *n* (%)	32 (6.7%)
**English fluency**, *n* (%)	
Very well	460 (97.0%)
Well	14 (3.0%)
Missing	5
**Speaks a language other than English**, *n* (%)	63 (13.3%)
**Marital status**, *n* (%)	
Currently married	367 (77.4%)
Widowed	2 (0.4%)
Divorced	11 (2.3%)
Separated	11 (2.3%)
Never married	83 (17.5%)
Missing	5
**Education attainment**, *n* (%)	
Less than high school diploma	2 (0.4%)
High school diploma or GED	49 (10.3%)
Some college, but no degree	84 (17.7%)
Associates degree	32 (6.8%)
Bachelor's degree	197 (41.6%)
Post-graduate degree	110 (23.2%)
Missing	5
**Employment status**, *n* (%)	
Employed full time	191 (40.3%)
Employed part time	90 (19.0%)
Self-employed	21 (4.4%)
Out of work and seeking opportunities	20 (4.2%)
Homemaker or stay at home parent	149 (31.4%)
Unable to work	3 (0.6%)
Missing	5
**Has childcare during COVID-19 pandemic**, *n* (%)	187 (39.5%)
**Household size**	
*N*	474
Mean (SD)	4.0 (1.2)
**EDPS total score**	
*N*	479
Mean (SD)	11.1 (6.1)
**EDPS interpretation**, ***n*** **(%)**	
Depression not likely (range: 0–8)	165 (34.4%)
Depression possible (range: 9–30)	314 (65.6%)

a Identifies Latino/a participants classified as multiracial.

### Experiences with mental healthcare services

Of the 479 participants, 27% (126/474) reported having spoken to a counselor, therapist, or other mental health professional since giving birth. About half (64/126) of participants reported that the encounter was virtual. On average, it had been 67 (*SD* = 82.5) days between the time they took the survey and the last time that they spoke with their therapist *via* virtual encounter. Virtual encounters happened *via* video (42/64; 66%), telephone (29/64; 45%), and text message (8/64; 13%). Among all the participants who had received postpartum mental health care (*n* = 126), the degree of satisfaction with virtual mental healthcare encounters in general (regardless of the nature of their postpartum care) was significantly lower among Latinas (*n* = 11) compared to non-Latina participants (*n* = 115; Table 2). In fact, 36% more Latinas disagreed or strongly disagreed with the statement about being satisfied with the quality of care received during virtual encounters compared to non-Latina participants.

**Table 2:** Participant satisfaction with virtual mental healthcare encounters, by ethnicity.

	**Latina participants**	**Non-Latina participants**	**Group difference**	***P*-value**
	**(*N* = 11)**	**(*N* = 115)**	**(Latina—Non)**	
**I am satisfied with the quality of care I received during these virtual interactions**, *n* (%)				0.01^a^
Strongly agree	3 (27.3%)	41 (35.7%)	−8.4%	
Agree	3 (27.3%)	63 (54.8%)	−27.5%	
Disagree	3 (27.3%)	7 (6.1%)	21.2%	
Strongly disagree	2 (18.2%)	4 (3.5%)	14.7%	

a Fisher exact *p*-value.

### Concerns regarding virtual mental healthcare

In the total sample, 88% of participants reported being open to consulting a mental health professional in the future. Among those open to future mental health services, 91% (382/418) reported they would consider virtual mental health services. The top concerns for accessing virtual mental healthcare were privacy (226/479; 47.2%), cost (222/479; 46.3%), time (130/479; 27.1%), and trust (128/479; 26.7%). Participants who were not interested in virtual mental healthcare encounters provided open-ended responses to further elaborate on their lack of interest. The following themes emerged from these qualitative responses: (1) preference for in-person therapy, (2) no perceived need for therapy, (3) not comfortable speaking to a therapist, and (4) lack of privacy at home.

#### Theme 1: Preference for in-person therapy

Participants stated a preference for in-person therapy. Participants stated that they would feel uncomfortable discussing sensitive topics in a virtual environment (Box 1, extracts 1–2). These participants perceived that face-to-face conversations would be the most appropriate way to share their emotions. There was also aversion to virtual therapy because of a perception that it would not be tailored to their unique situation. Participants doubted that virtual therapy could be delivered in a way that made it feel as “personal” as a face-to-face encounter (Box 1, extract 3). Finally, participants believed that virtual therapy would not be as effective as in-person treatment. For some, this perception was influenced by negative past experiences with virtual therapy (Box 1, extracts 4–5).

Box 1 **Theme 1—Preference for in-person therapy.**

1. 32-year-old White participant: “I have comfort issues regarding virtual sessions of that nature and would much rather speak face to face with a counselor or therapist.”

2. 40-year-old Asian participant: “I just prefer being in person while talking about intimate things.”

3. 23-year-old African American participant: “I like for it (therapy) to feel more personal and the only way to do that is in person for me.”

4. 27-year-old White participant: “It (virtual therapy) doesn't work for me.”

5. 32-year-old White participant: “I feel like virtually would not feel as effective for me.”

#### Theme 2: No perceived need for therapy

Participants reported that they were not interested in virtual therapy because they currently did not have a need. Participants stated that they were not experiencing symptoms of depression and anxiety or that they felt well-emotionally (Box 2, extracts 1–3). However, the EPDS scores of 6/19 participants stating no perceived need for therapy indicated possible depression. Some participants explained that if there were days when they were feeling down (which could be triggered by the COVID-19 pandemic), they already had coping strategies, which included reaching out to a spouse/partner and other family members, engaging in physical activity, eating healthy, meditating, and getting adequate sleep (Box 2, extracts 4–5).

Box 2 **Theme 2—No perceived need for therapy.**

1. 29-year-old White participant (EPDS score = 8; depression not likely): “I'm not feeling down or hopeless.”

2. 32-year-old White participant (EPDS score = 3; depression not likely): “I'm not suffering from baby blues or any other depression.”

3. 22-year-old Asian participant (EPDS score = 11; possible depression): “I am sure that I am feeling okay mentally and spiritually.”

4. 32-year-old Asian participant (EPDS score = 11; possible depression): “I have a few random days here and there of anxiety, but that is related to COVID and are rare. I just do some meditation and go to sleep and feel back to normal the next day.”

5. 27-year-old White participant (EPDS score = 4; depression not likely): “I am happy and know how to navigate and pull myself back up if I happen to fall into a slump. We eat well, regularly exercise, I take my children hiking 3 days a week and do yoga 5 days a week too. I have a strong family support system and love my in-laws.”

#### Theme 3: Not comfortable speaking to a therapist

Participants stated a disinterest in virtual therapy because they would feel uncomfortable. A few participants described experiencing shyness and social anxiety, regardless of whether interactions with others were in-person or virtual (Box 3, extracts 1–2). Other participants explained that they would be uncomfortable speaking about sensitive topics with someone they never met before (Box 3, extracts 3–5). They expressed a desire to meet the mental health professional face-to-face before transitioning to a virtual environment.

Box 3 **Theme 3—Not comfortable speaking to a therapist.**

1. 18-year-old Latina participant: “I wouldn't feel comfortable. I'm a very shy person.”

2. 39-year-old participant*: “I have pretty bad social anxiety so even if I do go to talk to someone ill most likely shut down at the time.”

3. 31-year-old White participant: “I would want to meet with someone in person first. I feel it is a better way of getting to know someone.”

4. 24-year-old White participant: “Not comfortable sharing sensitive information to someone I haven't seen.”

5. 23-year-old White participant: “It's hard to feel comfortable with someone you haven't met in real life.”

^*^No race or ethnicity reported.

#### Theme 4: Lack of privacy at home

Participants explained that they were not interested in virtual therapy because they did not have privacy at home. Some participants did not want others to find out they were speaking to a therapist (Box 4, extract 1). Other participants were worried about members of their household listening during phone calls and reading text message conversations with a therapist (Box 4, extract 2–3).

Box 4 **Theme 4—Lack of privacy at home.**

1. 24-year-old Latina participant: “I would not sure want anyone to know I am seeing a therapist.”

2. 27-year-old White participant: “I would be afraid I would get interrupted, other people could hear me.”

3. 35-year-old White participant: “Too worried about people breaking into that to get my info or chats.”

## Discussion

The mixed methods study supports that even though two-thirds of the U.S. sample of women reported possible PPD during the COVID-19 pandemic, only 27% had consulted a mental health professional. Of note, Latina women reported lower satisfaction with virtual therapy compared to non-Latina women. This finding is concerning given that the U.S. Latinx population is disproportionately affected by both COVID-19 (24) and PPD (25) compared to their White counterparts. The results support that even though U.S. residents are in the midst of a pandemic causing many healthcare services to shift to telehealth, one-quarter of the sample still reported hesitancy with virtual therapy. For those not interested in virtual therapy, primary reasons identified for apprehension included a preference for in-person therapy, no perceived need, uncomfortable with the idea of virtual therapy, and lack of privacy at home.

### Postpartum depression and therapy use

This study reported higher rates of possible PPD compared to previous U.S. studies (3). Through the self-reported Pregnancy Risk Assessment Monitoring System, the CDC (1) reports that 1 in 8 women experience PPD, whereas this study's results were closer to 6 in 8 women. Our increased rates might be explained by diagnostic clarity. Many cases of PPD typically go undiagnosed due to structural barriers as well as a lack of proper assessment (8, 21, 22). We used the EPDS, a scale that specifically assesses depression symptoms during the postpartum period. Another explanation is the stress associated with the COVID-19 pandemic, which has included exacerbated fear (26), isolation (27), and financial strain (28).

### Implications for practice

Only 27% of women indicated consulting a mental health professional during the postpartum period. This discrepancy in need and use necessitates an urgent solution to increase accessibility to mental health services as well as proper education of symptoms related to PPD. Of those that did receive mental health services, approximately half were done virtually *via* video, telephone or text message platforms. Since COVID-19 has shifted many services to virtual platforms, it is important to understand patient concerns. Consistent with pre-pandemic research (16), some participants identify a preference for in-person therapy. This preference seems to stem from perceived increased authenticity in person (16) and worries that virtual therapy is less effective (16, 29).

Participants identified that they would not engage with virtual therapy because they did not perceive a need for therapy. Interestingly, of the 19 women that listed this concern, six endorsed elevated levels consistent with possible depression on the EPDS. Previous studies have highlighted stigma and beliefs about motherhood as barriers to treatment (30). While the American College of Obstetricians and Gynecologists and the US Preventive Services Task Force have strongly recommended universal screening, there may also be an increased need for education of PPD and its treatment (31).

Participants stated they would not be comfortable with virtual therapy, and that they had concerns with a lack of privacy at home. The COVID-19 pandemic has changed home environments for many with an increase in adults working from home (32) and school-age children engaging in remote learning (33). There is evidence to support that women are taking on more care and responsibilities during the COVID-19 pandemic (9), which may be driving comfort and privacy concerns regarding virtual therapy compared to pre-pandemic times.

### Limitations and future directions

This study had several limitations. Because our sample was recruited through an online platform, it is likely that participants were more comfortable with technology and may have different views about data security and privacy. This study was cross-sectional, thus, opinions about virtual therapy may change over time as the population has more access to telehealth during the pandemic. Such limitations do not detract from the importance of the findings, which highlight the low utilization and trust in virtual mental health care by women who are currently using technology. Likely because of our recruitment methods, our sampling of women of color was relatively low (e.g., 7% of this sample was Latina v. 17% of the U.S. population is Latinx). The recruitment reach of the platform used for this study makes it difficult to target specific demographics; a longer study period could help to oversample specific groups (including postpartum women). Survey instruments were also available in English only, further limiting the generalizability of our findings to English-speaking women who are likely more acculturated. We recommend a future study comparing remotely recruited samples to locally recruited (and demographically targeted) samples in their acceptance of virtual mental health care. We also did not collect information about the presence of a diagnosed mental disorder; future studies could benefit from such contextual information.

Despite these limitations, the data from this study have important implications about the concept that virtual mental health care is a viable alternative to traditionally delivered care. To improve the approachability of virtual therapy, researchers and clinicians may employ user-centered design alongside patients and better understand how women intend to use virtual therapy for support (13).

## Conclusion

These results further support that rates of psychological distress, such as PPD, are elevated during the COVID-19 pandemic. Despite most of our sample reporting symptoms consistent with possible depression symptoms, only one-quarter have used psychotherapy services. While many participants were open to engaging in virtual therapy, primary concerns included preference for in-person services, lack of perceived need, discomfort with virtual therapy, and lack of privacy. We recommend that researchers and clinicians continue to find ways to make virtual therapy more approachable to the needs of women experiencing PPD.

## Data availability statement

The raw data supporting the conclusions of this article will be made available by the authors, without undue reservation.

## Ethics statement

The studies involving human participants were reviewed and approved by University of Washington Institutional Review Board. The patients/participants provided their written informed consent to participate in this study.

## Author contributions

CG and MR: guarantors. CG, MR, PA, and NG: study concept and design. CG, MR, MD, MJ, and FM-G: acquisition, analysis, or interpretation of data. CG, FM-G, MR, MD, and AD: drafting of the manuscript. CG, MR, PA, and NG: obtaining funding. All authors critical revision of the manuscript, had full access to the study data, take responsibility for the integrity of the complete work, and the final decision to submit the manuscript.

## Funding

This work was supported by the National Institutes of Mental Health (grant numbers P50MH115837, T32MH020021). The content is solely the responsibilities of the authors and does not necessarily represent the official views of the National Institutes of Health.

## Conflict of interest

The authors declare that the research was conducted in the absence of any commercial or financial relationships that could be construed as a potential conflict of interest.

## Publisher's note

All claims expressed in this article are solely those of the authors and do not necessarily represent those of their affiliated organizations, or those of the publisher, the editors and the reviewers. Any product that may be evaluated in this article, or claim that may be made by its manufacturer, is not guaranteed or endorsed by the publisher.
